# Heavy metals and trace elements in maternal blood and prevalence of congenital limb abnormalities among newborns: the Japan Environment and Children’s Study

**DOI:** 10.1265/ehpm.23-00366

**Published:** 2024-07-23

**Authors:** Atsuko Ikeda, Megasari Marsela, Chihiro Miyashita, Takeshi Yamaguchi, Yasuaki Saijo, Yoshiya Ito, Hiroyoshi Iwata, Sachiko Itoh, Mariko Itoh, Keiko Yamazaki, Naomi Tamura, Sumitaka Kobayashi, Reiko Kishi, the Japan Environment and Children’s Study Group

**Affiliations:** 1 Faculty of Health Sciences, Hokkaido University, Sapporo, Japan; 2 Center for Environmental and Health Sciences, Hokkaido University, Sapporo, Japan; 3 Department of Social Medicine, Asahikawa Medical University, Asahikawa, Japan; 4 Faculty of Nursing, Japanese Red Cross Hokkaido College of Nursing, Kitami, Japan; 5 Division of Epidemiological Research for Chemical Disorders, Research Center for Chemical Information and Management, National Institute of Occupational Safety and Health, Kawasaki, Japan

**Keywords:** Heavy metals, Trace elements, Congenital limb abnormalities, Prenatal exposure, Children

## Abstract

**Background:**

Heavy metals such as lead (Pb) and cadmium (Cd) have been associated with adverse pregnancy and developmental outcomes, including congenital abnormalities. This study investigated the association between exposure to heavy metals and trace elements during fetal life and congenital limb abnormalities in infants.

**Methods:**

This study is based on a prospective ongoing nationwide birth cohort from the Japan Environment and Children’s Study (JECS). The concentrations of Cd, Pb, mercury (Hg), selenium (Se), and manganese (Mn) were measured in maternal blood collected during the mid–late trimesters. Inclusion criteria were available from questionnaires filled in during pregnancy, including information about congenital limb abnormalities at birth or at one month. To examine the associations with limb anomalies and individual chemicals, logistic regression models were applied following log-transformation or division into quartiles of Cd, Pb, Hg, Se, and Mn concentrations. To assess the associations with the heavy metals and trace elements mixture, quantile g-computation was employed. All models were adjusted for age, maternal smoking history, maternal alcohol intake, history of smoking, and infant sex.

**Results:**

Data from 90,163 participants were included in the analysis, of whom 369 had congenital limb abnormalities in any of the collected information, and 89,794 had none. Among the 369 cases of congenital limb abnormalities, there were 185 and 142 cases of polydactyly and syndactyly, respectively. The median concentrations of Pb, Cd, Hg, Se, and Mn were 5.85, 0.66, 3.64, 168, and 15.3 ng/g, respectively. There were no associations between maternal blood concentrations of Pb [adjusted odd ratio = 0.83; 95% confidence interval = 0.61, 1.11], Cd [0.87; 0.68, 1.10], Hg [0.88; 0.73, 1.07], Se [1.07; 0.44, 2.59], and Mn [0.91; 0.64, 1.30] with congenital limb abnormalities. No significant association was observed between the mixture of heavy metals and trace elements [0.85; 0.72, 1.02] and any congenital limb abnormalities. Moreover, there was no association with all polydactylies and all syndactylies, or any type of abnormality as a subdivision.

**Conclusion:**

At the maternal exposure levels of Cd, Pb, Hg, Se, and Mn assessed in the present study, no association was identified with the risk of developing congenital limb abnormalities in children.

**Supplementary information:**

The online version contains supplementary material available at https://doi.org/10.1265/ehpm.23-00366.

## 1. Introduction

Congenital limb amputations and deficiencies are missing or incomplete limbs at birth and are mostly caused by primary intrauterine growth inhibition or secondary impairment due to intrauterine damage to normal embryonic tissue [1]. About 20–25% of cases occur as a complication of genetic disorders [2], whereas about 10–12% are associated with prenatal exposure to drugs and environmental toxicants, e.g., thalidomide [3].

Heavy metals and trace elements have been associated with adverse pregnancy and developmental outcomes, including congenital abnormalities [4–7]. There is evidence from animal studies that heavy metal exposure to Cd and the trace elements of Mn and Se can cause congenital limb abnormalities [8–12]. Meanwhile, there are a few human studies that have shown a positive association between Pb and congenital heart defects, as well as between Pb and Cd and cleft lip and palate, and neural tube defects [13–15]. However, human studies have yet to examine the positive correlation of heavy metals with congenital limb abnormalities [16, 17].

The Japan Environment and Children's Study (JECS) is one of the largest national birth cohorts in the world, with more than 100,000 mother–child pairs participating from 2011 to 2014 [18]. Previously, the JECS investigated the association of heavy metals and trace elements with congenital abdominal abnormalities and isolated lip/palate clefts, but no significant association was found [19, 20]. However, embryologically, the differentiation of limbs and abdominal organs is different, as limbs are derived from mesoderm, whereas abdominal organs are derived from endoderm. According to the JECS data, the prevalence of congenital limb abnormalities defined by the medical records at birth and one month of age is 26.8 per 10,000 pregnancies [21]. The aim of this study was to investigate the association between exposure to heavy metals and trace elements during fetal life and congenital limb abnormalities in infants using data transcribed from the JECS.

## 2. Methods

### 2.1 Study participants in the JECS

The participants in this study were pregnant women and their children participating in the JECS. Pregnant women were recruited during early pregnancy at obstetric facilities and/or local government offices in 15 Regional Centres across a wide geographical area in Japan between January 2011 and March 2014. The participating proportion was estimated to be about 45%, which corresponds to about 3% of newborns during that period [22]. Details of this study, including the population, data collection, sampling of the biological specimens, and contents of the administered questionnaire, were described previously [18, 21–23].

### 2.2 Ethical statement

The JECS protocol was reviewed and approved by the Ministry of the Environment’s Institutional Review Board on Epidemiological Studies and the Ethics Committees of all participating institutions (Appendix A) (Ethical Number: No. 100910001, and ethical project identification code of Hokkaido University Center for Environmental and Health Sciences: Kanken 19–117). All participants gave written informed consent for inclusion before they participated in the study. The study was conducted in accordance with the Declaration of Helsinki (sixth version) and other nationally valid regulations.

### 2.3 Definition of outcomes

Congenital limb abnormalities included six types of abnormalities [21]: Polydactyly of fingers (Q69, Q70.4), Syndactyly of fingers (Q70), Cleft hand (Q71.6), Polydactyly of toes (Q69, Q70.4), Syndactyly of toes (Q70), and Cleft foot (Q71.6) (World Health Organization (WHO) International Classification of Diseases 10th Revision (ICD-10)) in addition to other limb abnormalities (Table 1). The information on congenital limb abnormalities among offspring was transcribed primarily from the medical records at birth (Dr0m) or 1 month (Dr1m). The secondary collection confirmed diagnoses of congenital limb abnormalities with the physicians after the mother’s report by checking a box on a questionnaire when the child was 2 years of age. The questionnaires asked caregivers to fill in the names of any diseases with which children had been diagnosed by physicians. Then, the diseases were confirmed by the physician in charge of the diagnosis. As for the primary outcomes, any of the congenital limb abnormalities mentioned above were used. We further assessed the additional outcomes of all syndactyly and polydactyly cases in the hand and foot together. Each type of abnormality was also examined separately as a subdivision.

**Table 1:** Number of cases defined as congenital limb abnormalities

**Congenital limb abnormalities**	**ICD-10 code**	**Defined cases by ** **medical records**	**Defined cases by Disease ** **Information Registry**	**Total**
All congenital limb abnormalities		245	255	369
All polydactilies		159	113	185
All syndactilies		114	84	142

Upper limb				
Polydactyly of fingers	Q69, Q70.4	92	59	
Syndactyly of fingers	Q70	37	20	
Short fingers	Q74	N/A	8	
Cleft hand	Q71.6	5	4	
Other upper limb abnormalities		13	34	

Lower limb				
Polydactyly of toes	Q69, Q70.4	80	58	
Syndactyly of toes	Q70	89	67	
Short toes	Q74.2	N/A	3	
Cleft foot	Q72.7	7	2	
Other lower limb abnormalities		3	77	

N/A: not applicable

### 2.4 Selection flow of study participants

**Fig. 1:**
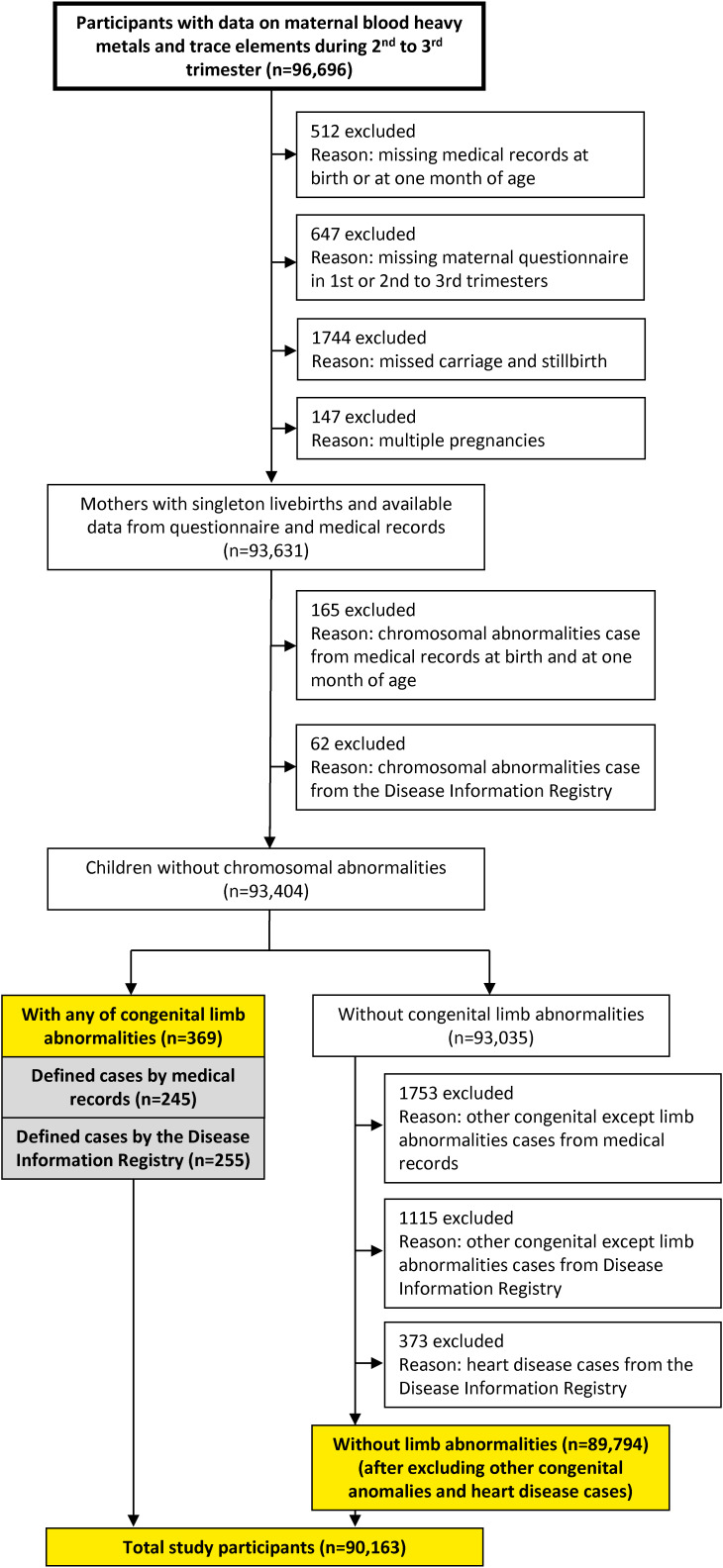
Flowchart of the selection of study participants

In this study, the JECS dataset jecs-ta-202190930 was used. The flowchart in Fig. 1 illustrates the selection of the study participants. The study participants were defined as those who met all of the following selection criteria and none of the exclusion criteria. The inclusion criteria were as follows: participants whose data of Cd, Pb, Hg, Se, and Mn levels (n = 96,696), data of medical records at birth or one month after birth (Dr0m1m), and data of the first trimester (MT1) or the second/third trimester (MT2) were available. Participants who experienced miscarriage, stillbirth, and multiple pregnancies were excluded, and the remaining number was 93,631. Participants with chromosomal abnormalities from the Medical Record Dr0m1m and the Disease Information Registry were further excluded, and the remaining number was 93,404. Among them, the total number of participants with any congenital limb abnormalities was 369. Participants without congenital limb abnormalities were subjected to the additional exclusion for congenital abnormalities other than limb abnormalities or heart disease identified in the Medical Record Dr0m1m and the Disease Information Registry (n = 89,794). Finally, the total number of study participants was 90,163 including those with any congenital limb abnormalities (n = 369) and without any congenital abnormalities (n = 89,794).

### 2.5 Measurement of the blood content of heavy metals and trace elements

Blood metal concentrations in the blood of pregnant women were assessed following previously described methods [23]. In brief, a 33 ml blood sample was collected from a peripheral vein during medical examinations performed in the second or third trimester. Samples were stored at −80 degrees Celsius until analysis. The samples were brought to room temperature and vortex-mixed before preparing aliquots. Quality control (QC), blank water, and blood samples (200 µl) were diluted 1:19 (v/v) in a dilution solution comprising 2% (v/v) butan-1-ol, 0.1% tetramethylammonium hydroxide, 0.5 g/L Polyoxyethylene octylphenyl ether, and 0.5 g/L ethylenediaminetetraacetic acid. The mixture was vortex-mixed again prior to analysis using inductively coupled plasma-mass spectrometry (ICP-MS). Samples outside the calibration range were reanalyzed following further dilution.

ICP-MS measurements were conducted using an Agilent 7700 ICP-MS (Agilent Technologies, Tokyo, Japan). Repeatability and intermediate precision were determined based on ISO 5725:1994 and 27148:2010 standards. Repeatability was further assessed through analysis of the reference standards and pooled QC samples, which was performed by a single operator using a single machine within one day, while intermediate precision was determined by continuous analysis of Seronorm™ Trace Element Whole Blood L-1 (REF 210105, Lot. 1003191, Sero AS, Billingstad, Norway). Assessment of QC samples was performed by multiple personnel using multiple machines over several days. The QC samples underwent the same procedure as whole blood samples and were analyzed twice in each analytical sequence. The Shewhart control chart (X-Rm chart) was utilized for day-to-day QC analysis following ISO 7870 standards. The repeatability and intermediate precision were found to be 1.6% and 2.5% for Hg, 0.82% and 1.2% for Pb, 1.7% and 3.5% for Cd, 3.4% and 1.4% for Mn, and 1.4% and 0.89% for Se, respectively, all expressed as relative standard deviations. All measured whole blood concentrations of Cd, Hg, Mn, Pb, and Se exceeded the detection limits of their respective methods (0.0234 ng/g, 0.049 ng/g, 0.522 ng/g, 0.129 ng/g, and 0.837 ng/g, respectively) [23].

### 2.6 Statistical analysis

The distribution of Cd, Pb, Hg, Se, and Mn among those with and without congenital limb abnormalities was examined using the Mann–Whitney U test. Spearman analysis was conducted to identify any correlations among Pb, Cd, Hg, Se, and Mn. Analyses of associations between individual exposure to Cd, Pb, Hg, Se, and Mn and limb abnormalities were performed by logistic regression. We constructed two exposure models: the first was a continuous model using log natural transformed metal concentrations, and the second was a quartile model with quartile 1 as a reference. The crude and adjusted odds ratios (ORs) with 95% confidence intervals (CIs) were calculated using the values of Cd, Pb, Hg, Se, and Mn introduced into the logistic model separately.

Quantile (Q) g-computation was employed to examine the partial and joint effects of the mixture of heavy metals and trace elements. Qg-computation involves the adaptation of the weighted quantile regression approach, and enhances causal inferential aspects using qg-computation, utilizing the following equation [24]:

$${\text{Y}}_{\text{i}}=\beta_{0}+{\Sigma^{\text{d}}}_{\text{j}=1}\beta_{\text{j}}{{\text{X}}^{\text{q}}}_{\text{ji}}+\varepsilon_{\text{i}}$$

Here, Y_i_ represents the health outcome for an individual i (i = 1, 2, 3…n), β_0_ denotes the model intercept, X^q^_ji_ is a quartile version of the j^th^ chemical exposure, ∑^d^_j=1_β_j_ represents the weighted quantile sum, estimating the combined effect of increasing every exposure in the metals and elements mixture by one quantile simultaneously, and ε_i_ is the error term. Qg-computation also enables the evaluation of the individual contributions of the mixture, while simultaneously estimating the positive or negative weight. The sum of the positive and negative weights is defined as 1.0. Each model was run for 500 iterations using bootstrapping.

For adjustment, covariates were introduced into the model using the forced entry method. Selection of covariates were based on the previous study, with covariates including maternal age (categorical), maternal smoking history (categorical), maternal alcohol intake (categorical), history of husband/partner (baby’s father) smoking (categorical), and infant sex (categorical) [20].

Statistical significance was set at p < 0.05 on both sides for a continuous model of logistic regression and qg-computation. Due to repeated testing for the quartile model of logistic regression, Bonferroni’s adjustment was adopted, and statistical significance was set at p < 0.05/3, which is 0.0167. The sample sizes of the cases defined by the Medical Records Dr0m1m and the Disease Information Registry were 245 and 255, respectively. With this sample size, the expected effect size was 0.25 when both α = 0.05 and β = 0.2 [25].

Statistical analyses were performed using SPSS software for Windows (version 26; IBM, Armonk, NY, USA), except for qg-computation. Qg-computation analysis was performed using the R (Version 4.2.3) package “qgcomp”.

## 3. Results

In this study, among the total study participants, 90,163 mother–child pairs were analyzed, of whom 369 had congenital limb abnormalities and 89,794 had no congenital limb abnormalities, other congenital anomalies, and heart disease, including the Medical Records Dr0m1m and Disease Information Registry (Table 1). Among the 369 cases of congenital limb abnormalities, there were 185 and 142 cases of polydactyly and syndactyly, respectively. The number of upper limb abnormalities, according to the Medical Records Dr0m1m and Disease Information Registry, included 92 and 59 cases of polydactyly of fingers, 37 and 20 cases of syndactyly of fingers, not applicable (N/A) and 8 cases of short fingers, 5 and 4 cases of cleft hand, and 13 and 34 cases of other upper limb abnormalities, respectively. The number of lower limb abnormalities according to the Medical Records Dr0m1m and Disease Information Registry included 80 and 58 cases of polydactyly of toes, 89 and 67 cases of syndactyly of toes, N/A and 3 cases of short toes, 7 and 2 cases of cleft foot, and 3 and 77 cases of other lower limb abnormalities, respectively. N/A in the short fingers and toes data means that the data were not applicable because no relevant questionnaire was available (Table 1).

Parent and child characteristics are shown in Table 2. A slightly larger proportion of mothers of children with congenital limb abnormalities fell within the maternal age range of 30 to <35 years old and over 35 years old compared to those without abnormalities. The distribution of parent characteristics among children with and without congenital limb abnormalities was similar, exhibiting no apparent differences. Children with congenital limb abnormalities displayed shorter gestational weeks, lower birth weights, shorter birth heights, and smaller birth chest circumference in comparison to those without congenital limb abnormalities.

**Table 2:** Parent and child characteristics

**Characteristics**		**Total**		**Without congenital limb abnormalities**		**All congenital limb abnormalities**	
		**n = 90163**	**100%**	**n = 89794**	**100%**	**n = 369**	**100%**
Mother							
Age	<25	9026	10.01%	9000	10.02%	26	7.05%
	25 to <30	24961	27.68%	24868	27.69%	93	25.20%
	30 to <35	31966	35.45%	31824	35.44%	142	38.48%
	35+	24204	26.84%	24096	26.83%	108	29.27%
	Missing data	6	0.01%	6	0.01%	0	0.00%
Prepregnancy BMI	<18.5	14460	16.04%	14406	16.04%	54	14.63%
	18.5–25	66090	73.30%	65819	73.30%	271	73.44%
	25+	9558	10.60%	9514	10.60%	44	11.92%
	Missing data	55	0.06%	55	0.06%	0	0.00%
Parity	Nulliparous	36981	41.02%	36823	41.01%	158	42.82%
	Multiparous	53136	58.93%	52925	58.94%	211	57.18%
	Missing data	46	0.05%	46	0.05%	0	0.00%
Marital status	Married	85583	94.92%	85229	94.92%	354	95.93%
	Single	3113	3.45%	3104	3.46%	9	2.44%
	Divorce	738	0.82%	735	0.82%	3	0.81%
	Widow	14	0.02%	14	0.02%	0	0.00%
	Missing data	715	0.79%	712	0.79%	3	0.81%
Education	≤12 years	32560	36.11%	32431	36.12%	129	34.96%
	≥13 years	56494	62.66%	56258	62.65%	236	63.96%
	Missing data	1109	1.23%	1105	1.23%	4	1.08%
Occupation	Fulltime, house business, temporal staff	33973	37.68%	33828	37.67%	145	39.30%
	Part time, others	20902	23.18%	20818	23.18%	84	22.76%
	Housewife	33904	37.60%	33769	37.61%	135	36.59%
	Unemployment	1249	1.39%	1244	1.39%	5	1.36%
	Missing data	135	0.15%	135	0.15%	0	0.00%
Smoking	Never	52142	57.83%	51936	57.84%	206	55.83%
	Quit before conception	21358	23.69%	21266	23.68%	92	24.93%
	Quit after conception	12068	13.38%	12023	13.39%	45	12.20%
	Smoking	4429	4.91%	4403	4.90%	26	7.05%
	Missing data	166	0.18%	166	0.18%	0	0.00%
Alcohol	Never	31149	34.55%	31011	34.54%	138	37.40%
	Quit	50026	55.48%	49828	55.49%	198	53.66%
	Almost none	1073	1.19%	1068	1.19%	5	1.36%
	Drink	7880	8.74%	7852	8.74%	28	7.59%
	Missing data	35	0.04%	35	0.04%	0	0.00%
Folate supplementation	Less than once/week	50629	56.15%	50421	56.15%	208	56.37%
	More than once/week	14280	15.84%	14210	15.83%	70	18.97%
	Everyday	25235	27.99%	25144	28.00%	91	24.66%
	Missing data	19	0.02%	19	0.02%	0	0.00%
Assisted Reproductive Technology	Yes	7579	8.41%	7536	8.39%	43	11.65%
	No	81850	90.78%	81526	90.79%	324	87.80%
	Missing data	734	0.81%	732	0.82%	2	0.54%
Maternal medical drug usage during pregnancy	Yes	79433	88.10%	79101	88.09%	332	89.97%
	No	9973	11.06%	9938	11.07%	35	9.49%
	Missing data	757	0.84%	755	0.84%	2	0.54%
Mode of delivery	Vaginal	73468	81.48%	73178	81.50%	290	78.59%
	C-section	16462	18.26%	16384	18.25%	78	21.14%
	Missing data	233	0.26%	232	0.26%	1	0.27%
Household Income	≤2 million	4739	5.26%	4722	5.26%	17	4.61%
	2 to <4	28928	32.08%	28816	32.09%	112	30.35%
	4 to <6	27453	30.45%	27337	30.44%	116	31.44%
	6 to <8	13151	14.59%	13091	14.58%	60	16.26%
	8 to <10	5368	5.95%	5353	5.96%	15	4.07%
	10+	3521	3.91%	3504	3.90%	17	4.61%
	Missing data	7003	7.77%	6971	7.76%	32	8.67%

Father							
Smoking	Never	24124	26.76%	24027	26.76%	97	26.29%
	Quit before conception	20790	23.06%	20699	23.05%	91	24.66%
	Quit after conception	2237	2.48%	2230	2.48%	7	1.90%
	Smoking	42354	46.97%	42181	46.98%	173	46.88%
	Missing data	658	0.73%	657	0.73%	1	0.27%
Education	≤12 years	39309	43.60%	39138	43.59%	171	46.34%
	≥13 years	49187	54.55%	48995	54.56%	192	52.03%
	Missing data	1667	1.85%	1661	1.85%	6	1.63%

Infant							
Child sex	Boys	44232	49.06%	44066	49.07%	166	44.99%
	Girls	45927	50.94%	45724	50.92%	203	55.01%
	Missing data	4	0.00%	4	0.00%	0	0.00%
Gestational week	≥37	86392	95.82%	86048	95.83%	344	93.22%
	<37	3733	4.14%	3708	4.13%	25	6.78%
	Missing data	38	0.04%	38	0.04%	0	0.00%

		Mean	±SD	Mean	±SD	Mean	±SD
Birth weight	n = 90076	3034.49	401.41	3034.78	401.05	2962.11	476.61
Birth height	n = 89858	48.98	2.16	48.98	2.16	48.56	2.72
Birth head circumference	n = 89701	33.21	1.45	33.21	1.45	33.10	1.57
Birth chest circumference	n = 89676	31.81	1.74	31.81	1.74	31.55	2.28

The distribution of Pb, Cd, Hg, Se, and Mn concentrations (µg/L) are shown in Table 3. Maternal Pb, Cd, Hg, Se, and Mn levels were detected in all samples (detection frequency 100%), and the median concentrations were 5.85, 0.66, 3.64, 168, and 15.3 µg/L, respectively. There were no significant differences in maternal Pb, Cd, Hg, Se, and Mn concentrations between infants with any and no congenital limb abnormalities. Although statistically significant, the correlation among the heavy metals and trace elements were weak (Spearman’s ρ = 0.028–0.292) (Supplementary Table S1).

**Table 3:** Distribution of Pb, Cd, Hg, Se, and Mn concentrations (ng/g)

	**Mean**	**SD**	**Min**	**25%**	**50%**	**75%**	**Max**	**p-value**
Total study participants, n = 90163								
Pb	6.34	2.86	1.20	4.70	5.85	7.33	110	
Cd	0.75	0.38	0.10	0.50	0.66	0.90	5.33	
Hg	4.20	2.49	0.18	2.54	3.64	5.20	58.8	
Se	170	20.3	82.8	156	168	182	976	
Mn	15.9	4.66	3.06	12.6	15.3	18.6	60.8	

All congenital limb abnormalities, n = 369								
Pb	6.27	2.86	2.25	4.62	5.76	7.21	30.8	0.339
Cd	0.74	0.37	0.19	0.50	0.66	0.87	2.92	0.811
Hg	4.08	2.31	0.55	2.52	3.56	5.14	14.6	0.402
Se	171	23.0	112	156	168	183	318	0.912
Mn	15.8	4.63	6.78	12.35	15.2	18.3	31.0	0.478

Without congenital limb abnormalities, n = 89794								
Pb	6.34	2.86	1.20	4.70	5.85	7.33	110	
Cd	0.75	0.38	0.10	0.50	0.66	0.90	5.33	
Hg	4.20	2.49	0.18	2.54	3.64	5.20	58.8	
Se	170	20.3	82.8	156	168	182	976	
Mn	15.9	4.66	3.06	12.6	15.3	18.6	60.8	

p-values were calculated by Mann-Whitney U test between participants all and without congenital limb abnormalities.

No association with maternal blood concentrations of individual heavy metals and trace elements was found when all congenital limb abnormalities (Pb [adjusted OR = 0.83; 95%CI = 0.61, 1.11], Cd [0.87; 0.68, 1.10], Hg [0.88; 0.73, 1.07], Se [1.07; 0.44, 2.59], and Mn [0.91; 0.64, 1.30]), all polydactylies (Pb [0.74; 0.49, 1.14], Cd [0.97; 0.69, 1.37], Hg [1.02; 0.78, 1.33], Se [1.47; 0.43, 5.06], and Mn [1.09; 0.66, 1.80]), and all syndactylies (Pb [0.75; 0.46, 1.21], Cd [0.82; 0.55, 1.21], Hg [0.77; 0.57, 1.05], Se [0.56; 0.13, 2.34], and Mn [1.01; 0.57, 1.78]) were assessed by either Dr0m1m or the Disease Information Registry in linear regression models (Table 4). Finally, as a subdivision, there was a significant association between Hg and low OR (95% CI) of syndactyly of toes in the Dr0m1m data (OR, 0.59; 95% CI, 0.40–0.87, p = 0.007) (Supplementary Table S2). Similarly, there was a significant association between Hg and low OR (95% CI) of syndactyly of fingers in the Disease Information Registry data (OR, 0.42; 95% CI, 0.19–0.94, p = 0.035) (Supplementary Table S3). However, these associations were not observed in the categorical models (Supplementary Table S2 and Supplementary Table S3). Due to the small number of cases, we did not examine associations for short fingers and toes, cleft hand and foot, and other upper and lower limb abnormalities (Supplementary Table S2 and Supplementary Table S3).

**Table 4:** Pb, Cd, Hg, Se, and Mn exposure and congenital limb abnormalities

**Variables**	**Continuous model**				**Categorical model**																

					**1st quartile**		**2nd quartile**					**3rd quartile**					**4th quartile**				

	**adjusted OR**	**95%CI**		**p-value**	**cases/non-cases (n)**		**cases/non-cases (n)**	**adjusted OR**	**95%CI**		**p-value**	**cases/non-cases (n)**	**adjusted OR**	**95%CI**		**p-value**	**cases/non-cases (n)**	**adjusted OR**	**95%CI**		**p-value**
All congenital limb abnormalities (n = 369)																					
Pb	0.83	0.61	1.11	0.207	105/22652	ref	41/22350	0.82	0.62	1.10	0.184	58/22540	0.90	0.68	1.19	0.475	35/22252	0.76	0.57	1.02	0.066
Cd	0.87	0.68	1.10	0.242	89/22706	ref	43/22393	1.05	0.79	1.40	0.740	53/22424	1.07	0.80	1.42	0.663	42/22271	0.79	0.58	1.09	0.150
Hg	0.88	0.73	1.07	0.198	94/22499	ref	47/22387	1.01	0.76	1.34	0.942	42/22437	0.94	0.70	1.25	0.662	51/22471	0.91	0.68	1.22	0.540
Se	1.07	0.44	2.59	0.877	97/22536	ref	44/22880	0.96	0.72	1.27	0.765	35/23073	0.85	0.63	1.13	0.259	52/21305	0.99	0.74	1.31	0.916
Mn	0.91	0.64	1.30	0.618	102/22673	ref	42/22981	0.91	0.68	1.20	0.495	46/22274	0.90	0.68	1.20	0.469	47/21866	0.88	0.66	1.18	0.384

All polydactilies (n = 185)																					
Pb	0.74	0.49	1.14	0.172	51/22652	ref	36/22350	0.80	0.53	1.21	0.300	37/22540	1.12	0.77	1.63	0.560	30/22252	0.67	0.43	1.03	0.067
Cd	0.97	0.69	1.37	0.876	47/22706	ref	44/22393	0.89	0.59	1.35	0.594	35/22424	1.07	0.72	1.60	0.739	33/22271	0.80	0.52	1.24	0.319
Hg	1.02	0.78	1.33	0.879	45/22499	ref	38/22387	1.05	0.69	1.57	0.834	42/22437	0.93	0.61	1.41	0.718	26/22471	1.10	0.74	1.65	0.633
Se	1.47	0.43	5.06	0.538	54/22536	ref	36/22880	0.80	0.54	1.19	0.265	28/23073	0.63	0.41	0.96	0.031	35/21305	1.00	0.68	1.47	0.989
Mn	1.09	0.66	1.80	0.728	50/22673	ref	37/22981	0.83	0.55	1.25	0.378	32/22274	0.94	0.63	1.40	0.759	37/21866	0.97	0.65	1.45	0.896

All syndactilies (n = 142)																					
Pb	0.75	0.46	1.21	0.235	39/22652	ref	86/22350	0.93	0.59	1.46	0.749	96/22540	0.94	0.60	1.48	0.803	82/22252	0.75	0.47	1.22	0.252
Cd	0.82	0.55	1.21	0.312	30/22706	ref	97/22393	1.44	0.90	2.29	0.127	102/22424	1.11	0.68	1.82	0.674	81/22271	0.99	0.59	1.67	0.977
Hg	0.77	0.57	1.05	0.098	36/22499	ref	96/22387	1.04	0.66	1.65	0.853	90/22437	1.15	0.73	1.79	0.553	89/22471	0.70	0.42	1.16	0.166
Se	0.56	0.13	2.34	0.424	43/22536	ref	95/22880	0.82	0.52	1.27	0.365	85/23073	0.62	0.39	1.01	0.053	92/21305	0.84	0.54	1.32	0.455
Mn	1.01	0.57	1.78	0.982	36/22673	ref	93/22981	1.02	0.65	1.62	0.921	89/22274	0.92	0.57	1.48	0.732	85/21866	1.10	0.69	1.74	0.699

The Odds Ratios were calculated using the natural log transformation of the metal or element in the continuous model, while in the categorical model, the metal or element was divided into quartiles, with the 1st quartile serving as the reference.

Adjusted for maternal age (categorical), maternal smoking (categorical), maternal alcohol intake (categorical), paternal smoking (categorical), infant sex.

The quartile model’s p-value is defined as 0.05/3 = 0.0167 (Bonferroni’s adjustment).

No significant association was identified between the mixture of heavy metals and trace elements and all congenital limb abnormalities [adjusted OR = 0.85; 95%CI = 0.72, 1.02], all polydactylies [0.92; 0.72, 1.17], and all syndactylies [0.83; 0.63, 1.11] (Table 5). Most heavy metals and trace elements demonstrated a negative direction in scaled effect sizes, as indicated by the qg-computation results (Supplementary Fig. S1).

**Table 5:** Associations between limb abnormalities and mixture exposure of Pb, Cd, Hg, Se, Mn.

	**adjusted OR**	**95%CI**		**p-value**	**Sum of positive coefficient**	**Sum of negative coefficient**
All congenital limb abnormalities	0.85	0.72	1.02	0.073	0	−0.16
All polydactylies	0.92	0.72	1.17	0.497	0.03	−0.12
All syndactylies	0.83	0.63	1.11	0.207	0.02	−0.20

The adjusted odds ratio are interpreted as the effect on limb abnormalities of increasing every one quantile unit of mixture exposure of Pb, Cd, Hg, Se, and Mn in quantile g-computation.

Adjusted for maternal age (categorical), maternal smoking (categorical), maternal alcohol intake (categorical), paternal smoking (categorical), infant sex.

## 4. Discussion

In the present study, no statistically significant associations were observed between individual concentrations of heavy metal and trace element maternal blood concentrations and all congenital limb abnormalities, all polydactylies, and all syndactylies. Most of the heavy metals and trace elements in the mixture analysis exhibited an inverse direction, although without reaching significance. Although there were some statistically significant associations in quartile models, there was no clear dose–response trend; therefore, some differences could be chance findings due to multiple testing. The results indicated that maternal Pb, Cd, Hg, Se, and Mn concentrations at the present levels were not a definite risk factor for congenital limb abnormalities in the offspring.

Direct comparison of results between previous studies and the present study is difficult because there is a limited number of studies assessing the effects of heavy metals and trace elements in maternal blood. In North Carolina, a human study was conducted to evaluate the association between metal concentrations in private well water and birth defect prevalence, including limb reduction [17]. Individual exposure was designated as the average metal concentrations in the census tract encompassing the geocoded maternal residence. No association was observed between congenital limb abnormalities and metal exposure [17]. In another study, based on the classification of exposure to emissions from municipal solid waste incinerators, data from exposed (n = 194) and unexposed (n = 2678) settlement populations were used to evaluate the relative risks of congenital abnormalities. The rate of congenital abnormalities, including limb abnormalities, was not significantly higher in exposed communities than in unexposed communities [26].

We did not observe an association between maternal exposure to individual and a mixture of Pb, Cd, Se, or Mn and the risk of congenital limb abnormalities in offspring in Japan’s large-scale nationwide birth cohort. None of the participants exceeded the guideline levels for these elements. However, these findings may not translate into large populations, as the exposure levels in our cohort were relatively low compared to other countries [23].

We found an inverse association between Hg and the syndactyly of toes or fingers, which means higher levels of Hg were associated with reduced limb abnormalities. Asian countries, including Japan, are known to have higher Hg exposure than Western countries [27]. In 1961, fish in Minamata Bay registered alarming Hg levels exceeding 10 µg/g, and the inhabitants living in the coastal area of the Yatsushiro Sea were toxically affected by methylmercury [28]. The methylmercury caused fetus poisoning via the placenta, called congenital Minamata disease. The observed initial symptoms of the disease were mental retardation, primitive reflexes, coordination disturbance, dysarthria, growth disorder, chorea-athetosis, hypersalivation, and limb deformation [29]. The average hair mercury concentration of 102 inhabitants of Tsunagi Town near Minamata City was 41.2 µg/g in 1960 [30]. Fortunately, from 1977 to 1990, sediment removal operations were conducted, resulting in a subsequent decrease in Hg levels. Then, in 1988, Hg levels were measured from the red blood cells of the inhabitants of Tsunagi Town, and these levels fell between the means of islanders on Suwanosejima Island (76.4 ng/g for males and 50.3 ng/g for females) and Takarajima Island (25.4 ng/g for males and 20.0 ng/g for females) [30]. These blood mercury levels were not higher than in other populations in Japan at that time [30] and were considerably lower than the tolerable methylmercury intake according to the Japan Food Safety Commission (44 µg/L, which is equivalent to 41.9 ng/g of the total Hg concentration in blood converting the value by 1:1.0506 [31]) [32]. In the present study, the median Hg concentration in maternal blood was 3.64 ng/g, which is fifty-fold lower than in early Minamata disease cases—the total hair Hg was 41.2 µg/g, which can be converted to 164.8 ng/g according to the blood level ratio of 250:1 reported by WHO in 1990 [33]. No adverse health effects are anticipated from this Hg level. However, this level is still higher than that found in most Western countries [27]. This difference may be explained by the amount of fish consumption since the Japanese generally consume more fish and shellfish than people in other countries [34]. In Japan, fish/seafood, especially pelagic fish, is an important exposure source for Hg, and at the same time, fish is known to be a good source of nutrition, including long-chain polyunsaturated fatty acids [35]. The positive effects of fish consumption may have been confounded by the reduced ORs of congenital limb abnormalities in the present study. As a result, our findings provide assurance that the current Hg levels pose no increased risk of congenital limb abnormalities.

The United States Center for Disease Control (CDC) recommends follow-up blood Pb tests for pregnant women whose blood Pb levels exceed 5 µg/dL (equivalent to 47.6 ng/g) and taking action to reduce exposure to Pb sources [36]. The CDC sets the action levels of Cd and Se at 5 µg/L and 500 µg/L, respectively [37]; however, action levels are not available for Mn [23]. Future confirmation may be needed in areas where higher exposure is anticipated. In addition, the critical window for limb abnormalities in fetal development occurs during early pregnancy, typically before the 12^th^ week of gestation. It is also important to consider the half-life when assessing the impact of heavy metals on fetal development. Half-life is the amount of time it takes for half of the substance to be eliminated from the body. The half-life of Pb, Hg, and Mn in the blood is estimated to be 28–36 days [38], 60–90 days [39], and less than 74 days [40], respectively. The half-life of Cd is typically measured in the kidneys but is similar to that estimated in blood, ranging from 6–38 years [41]. As for Se, the half-life of the rapid phase is 1–3 days, depending on the compound ingested, and that of the slow phase is 30–110 days [42]. If the half-life of a substance is long enough, there is a lower likelihood of misclassification, meaning that the measurement of exposure is less likely to be inaccurate due to the substance remaining in the body for an extended period of time. Therefore, as an implication for future research, it is crucial to measure exposure to Cd, Pb, Hg, Se, and Mn during this early phase of pregnancy.

A strength of this study is that the baseline data on early pregnancy in participants were prospectively collected using a nationwide cohort study, which has a large sample size and covers a considerable geographical proportion of Japan. The JECS has collected more than 100,000 records of mother–infant pairs, where Pb, Cd, Hg, Se, and Mn exposure in maternal blood was detected at a comprehensive detection rate. Despite excluding certain participants for the analysis in this study, we still retained a substantial number of participants, constituting 93.2% of the baseline JECS participants. The demographic characteristics of our study participants, i.e. the mother’s age, parent’s smoking, maternal alcohol intake, income, and children sex, were comparable to those of the original JECS population [22]. The selected characteristics of the mothers and children in the JECS were comparable with those obtained in Japan’s 2013 Vital Statistics Survey. Therefore, the JECS results, and therefore the results of this study, could be extrapolated to the Japanese general population [22]. Hence, we can assert the generalizability and capacity to extrapolate the results of our current study to the Japanese population. Cases of congenital limb abnormalities were identified from hospital records by obstetricians, which minimized miscalculation bias. Maternal blood Pb, Cd, Hg, Se, and Mn levels were determined by reliable and validated methods (IPC-MS) with high repeatability and precision [23].

Nevertheless, our study has some limitations. Maternal blood was transcribed from the mid–late trimesters of pregnancy, whereas the critical window of the embryogenesis period was in the first trimester of pregnancy. Case overlaps by Dr0m1m and the Disease Information Registry were limited. Dr0m1m data included all births and missing data due to insufficient information, which led to misclassification (results toward null). On the other hand, the Disease Information Registry has been confirmed by clinicians’ diagnoses, so the cases are highly reliable; however, missing data due to unreported cases from mothers and voluntary withdrawal before children reached a certain age resulted in underestimations of the actual number of cases. Even though similar results of no association were found with both case definitions, by Dr0m1m and the Disease Information Registry up to 3 years of age, the results were reliable. In this study, data on the history of congenital limb abnormalities of the participating parents were not provided. Unobserved potential confounders may also be associated with maternal Pb, Cd, Hg, Se, and Mn exposure and congenital limb abnormalities.

To our knowledge, this study is the first to reveal no significant association between maternal in-utero exposure to Pb, Cd, Hg, Se, and Mn and the prevalence of congenital limb abnormalities in a large cohort of the Japanese population. However, it is possible that maternal Pb, Cd, Hg, Se, and Mn levels have adverse effects on fetal development and subsequent health in children, including neurodevelopmental delays, lower birth weight, and smaller head circumference, as previously shown in other JECS cohorts [43, 44]. Further studies with follow-up in children are necessary to investigate the effects of maternal blood Pb, Cd, Hg, Se, and Mn levels on offspring after birth.

## Supplementary information

**Additional file 1:** **Supplementary Figure S1.** The results of quantile g-computation. The bars in the figure represent positive and negative weights, indicating the partial contribution of heavy metals and trace elements in the mixture to congenital limb abnormalities. The concentrations of heavy metals and trace elements in blood were natural log transformed. Adjusted for maternal age (categorical), maternal smoking (categorical), maternal alcohol intake (categorical), paternal smoking (categorical), infant sex. **Supplementary Table S1.** Spearman correlation analysis results among Pb, Cd, Hg, Se, Mn. **Supplementary Table S2.** Pb, Cd, Hg, Se, Mn exposure and congenital limb abnormalities defined by medical records. **Supplementary Table S3.** Pb, Cd, Hg, Se, Mn exposure and congenital limb abnormalities defined by Disease Information Registry.

## Declarations

### Availability of data and materials

Data are unsuitable for public deposition due to ethical restrictions and the legal framework of Japan. It is prohibited by the Act on the Protection of Personal Information (Act No. 57 of 30 May 2003, amendment 9 September 2015) to publicly deposit data containing personal information. Ethical Guidelines for Medical and Health Research Involving Human Subjects enforced by the Japan Ministry of Education, Culture, Sports, Science, and Technology and the Ministry of Health, Labor, and Welfare also restrict the open sharing of epidemiological data. All inquiries about access to data should be sent to: jecs-en@nies.go.jp. The person responsible for handling inquiries sent to this e-mail address is Dr. Shoji F. Nakayama, JECS Programme Office, National Institute for Environmental Studies.

## Appendix A. Members of the Japan Environment and Children’s Study (JECS) Group 2023

The authors also extend their sincere appreciation to the JECS Group as of 2023: Michihiro Kamijima (principal investigator, Nagoya City University, Nagoya, Japan), Shin Yamazaki (National Institute for Environmental Studies, Tsukuba, Japan), Yukihiro Ohya (National Center for Child Health and Development, Tokyo, Japan), Reiko Kishi (Hokkaido University, Sapporo, Japan), Nobuo Yaegashi (Tohoku University, Sendai, Japan), Koichi Hashimoto (Fukushima Medical University, Fukushima, Japan), Chisato Mori (Chiba University, Chiba, Japan), Shuichi Ito (Yokohama City University, Yokohama, Japan), Zentaro Yamagata (University of Yamanashi, Chuo, Japan), Hidekuni Inadera (University of Toyama, Toyama, Japan), Takeo Nakayama (Kyoto University, Kyoto, Japan), Tomotaka Sobue (Osaka University, Suita, Japan), Masayuki Shima (Hyogo Medical University, Nishinomiya, Japan), Seiji Kageyama (Tottori University, Yonago, Japan), Narufumi Suganuma (Kochi University, Nankoku, Japan), Shoichi Ohga (Kyushu University, Fukuoka, Japan), and Takahiko Katoh (Kumamoto University, Kumamoto, Japan).
