# Supplementary material for: Heavy metals and trace elements in maternal blood and prevalence of congenital limb abnormalities among newborns: the Japan Environment and Children’s Study
Source: Environ Health Prev Med. 2024 Jul 23;29:36. doi: 10.1265/ehpm.23-00366 (PMC11273044; doi:10.1265/ehpm.23-00366)
Supplement: Supplementary file 1 — Additional file 1: Supplementary Figure S1. The results of quantile g-computation. The bars in the figure represent positive and negative weights, indicating the partial contribution of heavy metals and trace elements in the mixture to congenital limb abnormalities. The concentrations of heavy metals and trace elements in blood were natural log transformed. Adjusted for maternal age (categorical), maternal smoking (categorical), maternal alcohol intake (categorical), paternal smoking (categorical), infant sex. Supplementary Table S1. Spearman correlation analysis results among Pb, Cd, Hg, Se, Mn. Supplementary Table S2. Pb, Cd, Hg, Se, Mn exposure and congenital limb abnormalities defined by medical records. Supplementary Table S3. Pb, Cd, Hg, Se, Mn exposure and congenital limb abnormalities defined by Disease Information Registry. [file ehpm-29-036-s001.docx]

**Heavy metals and trace elements in maternal blood and prevalence of congenital limb abnormalities among newborns: The Japan Environment and Children's Study**

Atsuko Ikeda 1,2#,*, Megasari Marsela1,#, Chihiro Miyashita2, Takeshi Yamaguchi2, Yasuaki Saijo3, Yoshiya Ito4, Hiroyoshi Iwata2, Sachiko Itoh2, Mariko Itoh2, Keiko Yamazaki2, Naomi Tamura2, Sumitaka Kobayashi2,5, Reiko Kishi2, the Japan Environment and Children’s Study Group

^1^Faculty of Health Sciences, Hokkaido University, Sapporo, Japan

^2^Center for Environmental and Health Sciences, Hokkaido University, Sapporo, Japan

^3^Department of Social Medicine, Asahikawa Medical University, Asahikawa, Japan

^4^Faculty of Nursing, Japanese Red Cross Hokkaido College of Nursing, Kitami, Japan

^5^Division of Epidemiological Research for Chemical Disorders, Research Center for Chemical Information and Management, National Institute of Occupational Safety and Health, Kawasaki, Japan

^#^Equally contributed

**Supplementary Figure S1.** The results of quantile g-computation. The bars in the figure represent positive and negative weights, indicating the partial contribution of heavy metals and trace elements in the mixture to congenital limb abnormalities. The concentrations of heavy metals and trace elements in blood were natural log transformed. Adjusted for maternal age (categorical), maternal smoking (categorical), maternal alcohol intake (categorical), paternal smoking (categorical), infant sex.


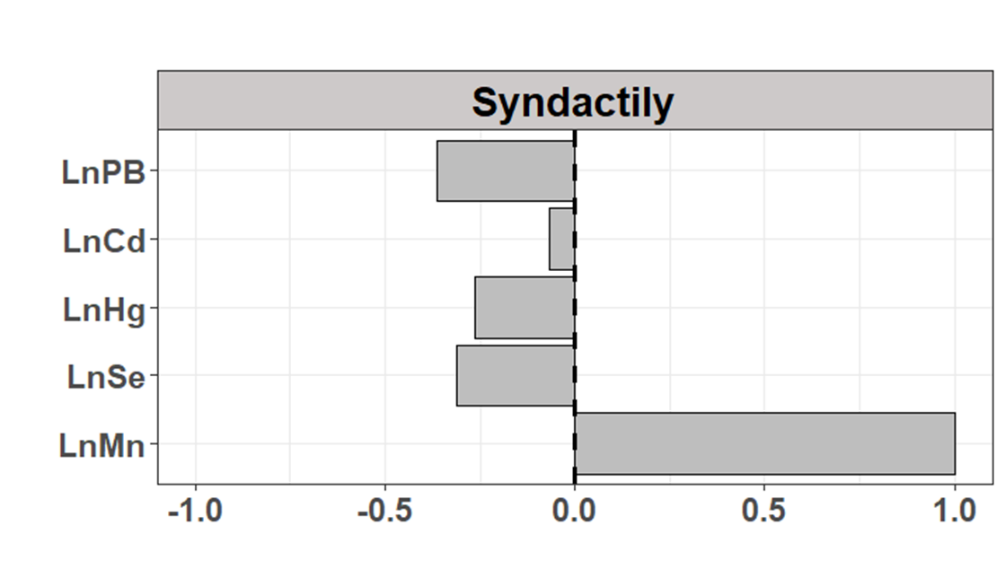

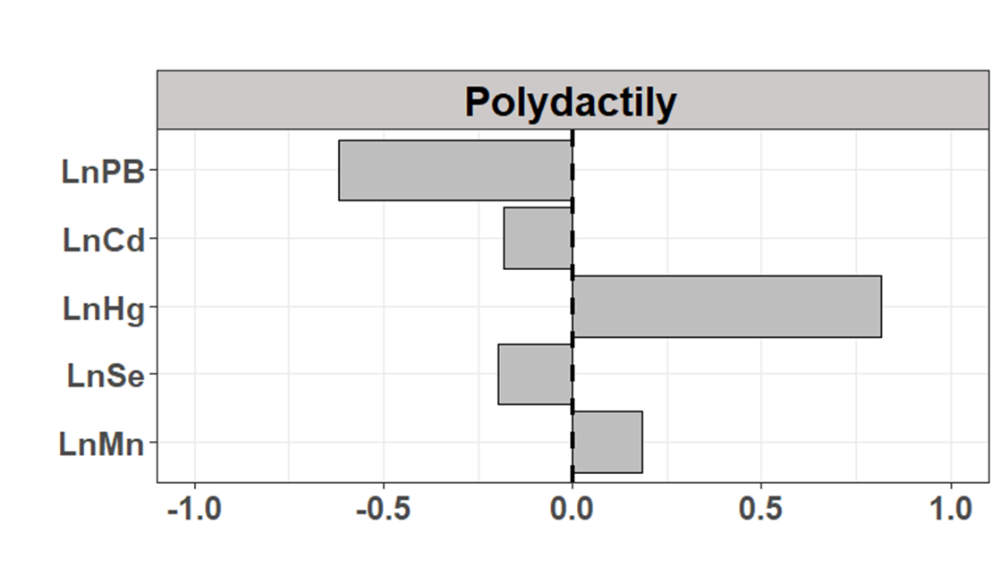

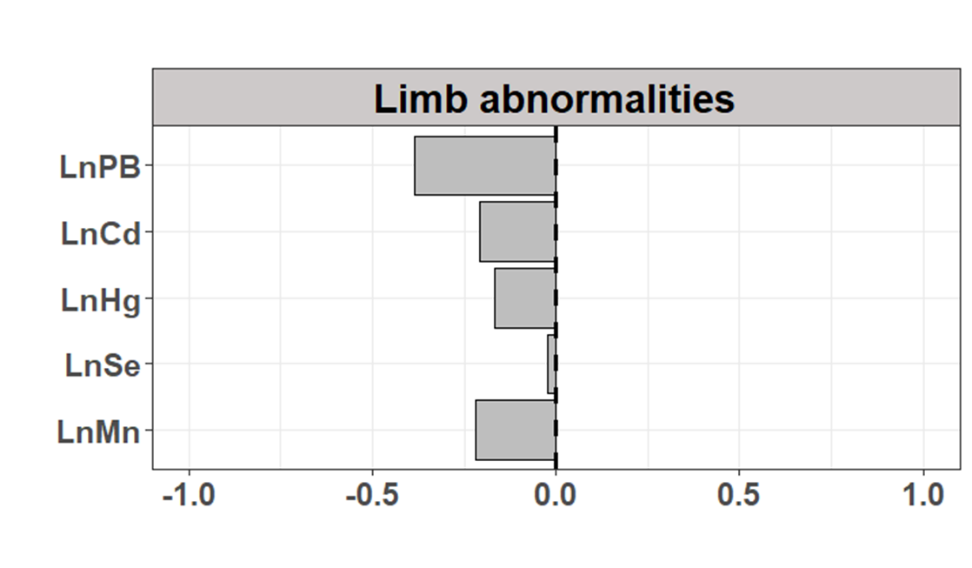


| **Supplementary Table S1.** Spearman correlation analysis results among Pb, Cd, Hg, Se, Mn | | | | | | | | | |
| --- | --- | --- | --- | --- | --- | --- | --- | --- | --- |
|  | **Correlation Coefficient** | | | | |  |  |  |  |
|  | **Pb** | **Cd** | **Hg** | **Se** | **Mn** |  |  |  |  |
| Pb | 1 |  |  |  |  |  |  |  |  |
| Cd | 0.248** | 1 |  |  |  |  |  |  |  |
| Hg | 0.092** | 0.053** | 1 |  |  |  |  |  |  |
| Se | 0.083** | 0.028** | 0.292** | 1 |  |  |  |  |  |
| Mn | 0.087** | 0.257** | -0.006 | 0.031** | 1 |  |  |  |  |
| ** Correlation is significant at the 0.01 level (2-tailed). | | | | | |  |  |  |  |
